# Management of Pediatric Parapneumonic Pleural Effusion: Interventional Versus Conservative Approaches in an 11-Year Retrospective Cohort

**DOI:** 10.3390/jcm15093310

**Published:** 2026-04-27

**Authors:** Bernat Servitje-Verdaguer, Romina Conti-Degiorgis, Roser Ayats-Vidal, Anna Gelman-Bagaria

**Affiliations:** 1 Department of Pediatrics, Parc Taulí University Hospital, 08208 Sabadell, Catalonia, Spain; 2 Parc Taulí Research and Innovation Institute (I3PT-CERCA), 08208 Sabadell, Catalonia, Spain; 3 Department of Pediatric Infectiology, Parc Taulí University Hospital, 08208 Sabadell, Catalonia, Spain; 4 Department of Pediatric Pneumology, Sant Joan de Déu University Hospital, 08950 Esplugues de Llobregat, Catalonia, Spain; 5 Department of Pediatric Hospitalization, Parc Taulí University Hospital, 08208 Sabadell, Catalonia, Spain

**Keywords:** parapneumonic pleural effusion, pediatric pneumonia, complicated pneumonia, empyema, pleural drainage, conservative treatment

## Abstract

**Background**: Parapneumonic pleural effusion (PPE) remains a relevant complication of pediatric pneumonia, with a substantial burden of morbidity, particularly in complicated forms. Optimal management strategies remain debated, with a recent shift toward more conservative approaches. Contemporary data on epidemiology, management practices, and outcomes are therefore needed. **Methods**: We conducted a retrospective cohort study of children admitted to our center with radiologically confirmed PPE between 2015 and 2025. Two study phases were defined to reflect the progressive shift in clinical practice: an interventional-prone period, in which complicated PPE (cPPE) was systematically drained, and a conservative period, in which drainage was reserved for patients with clinical deterioration. Data were compared between periods, and risk factors associated with pleural drainage during the conservative period were analyzed. **Results**: A total of 122 children with PPE were included (median age 4.1 years, 50% female), of whom 62.3% had cPPE. Pleural drainage was performed more frequently during the interventional period (55% vs. 24%). Patients managed during the conservative period had shorter duration of intravenous antibiotic therapy, shorter hospital stays, and faster radiological resolution, adjusting for disease severity. Within the conservative period, patients requiring pleural drainage (24.4%) had greater clinical and radiological severity, including higher rates of respiratory support and need for intensive care. **Conclusions**: In this cohort, the shift from a predominantly invasive to a more conservative strategy was not associated with worse clinical outcomes after adjusting for baseline severity. Pleural drainage was mainly reserved for patients with greater clinical compromise. These findings support a severity-guided approach to pleural drainage in pediatric PPE, in which conservative management with medical therapy alone may be safely considered in appropriately selected cases.

## 1. Introduction

Parapneumonic pleural effusion (PPE) remains one of the most common and challenging complications of pediatric community-acquired pneumonia [1,2,3,4]. Even though its overall incidence has declined following widespread pneumococcal vaccination [5,6,7,8], many regions have reported a paradoxical increase in the burden of PPE [9,10,11,12], particularly in the form of complicated PPE (cPPE) [11,12]. This trend highlights the persistent clinical relevance of pediatric PPE and the need for updated data on its epidemiology, management strategies, and outcomes.

The clinical spectrum of PPE ranges from small, uncomplicated effusions requiring only antibiotic therapy to large empyemas associated with respiratory complications and worsened outcomes [13,14,15]. Early identification of children at risk for cPPE is essential but remains challenging, as many proposed risk factors lack consistency and clinical applicability [13,16,17].

Management strategies for pediatric PPE continue to evolve [3,18,19,20,21,22]. While an interventional approach including pleural drainage, intrapleural fibrinolysis, and surgical procedures was once considered the paradigm, recent years have seen a shift toward more conservative strategies relying on intravenous antibiotics [18,21,22]. However, considerable variability between centers persists, and comparative data evaluating the impact of different management approaches on clinical outcomes remain limited [18,23].

In this framework, this study aimed to (1) compare clinical characteristics, management strategies, and outcomes across two periods reflecting a progressive shift from predominantly interventional to predominantly conservative management and (2) identify clinical and radiological factors associated with pleural drainage during the conservative period.

## 2. Materials and Methods

### 2.1. Study Design and Participants

We conducted an 11-year retrospective cohort study at our tertiary referral center (Parc Taulí University Hospital), including all patients aged 0–17 years admitted with radiologically confirmed PPE between January 2015 and December 2025. Exclusion criteria comprised pleural effusion of non-infectious etiology and severe underlying conditions. Data were retrieved from a prospectively maintained institutional database and complemented by review of electronic medical records when required. The study protocol was approved by the hospital’s Clinical Research Ethics Committee.

### 2.2. Definitions

PPE was defined as pleural effusion associated with community-acquired pneumonia identified by chest imaging (ultrasound, radiograph, or computed tomography). cPPE was defined according to standard biochemical criteria for empyema (pH < 7,0, purulent appearance, and/or positive microbiological identification) and/or radiological criteria for loculated effusion (evidence of septations) [24]. Radiologic resolution was defined as the time from admission to the first available chest radiograph without PPE-related pathologic findings, according to interpretation by a specialist pediatric pulmonologist.

Management approaches were categorized as interventional (pleural drainage, intrapleural fibrinolysis, or surgical procedures such as video-assisted thoracoscopic surgery) or conservative consisting of medical therapy alone (antibiotic therapy, analgesia, respiratory rehabilitation).

### 2.3. Study Intervention and Data Collection

To evaluate temporal changes in clinical practice, the study was divided into two predefined periods with differentiated therapeutic strategies at our institution. Although no abrupt change in management occurred, but rather a gradual transition, these two periods successfully reflected a shift toward a more conservative strategy.

An interventional period (2015–2022) was defined, characterized by a lower threshold for invasive procedures, in which both clinical compromise and the presence of cPPE were generally considered indications for pleural drainage. This was followed by a conservative period (2023–2025), marked by increasing reliance on medical therapy alone, with pleural drainage progressively reserved for patients with clinical deterioration. These two periods were used to assess whether changes in clinical practice were associated with differences in outcomes.

Demographic data included age, sex, weight, height, vaccination status, prior medical conditions, and concomitant respiratory infection. Clinical variables at presentation included fever, respiratory symptoms, dyspnea, retractions, abdominal pain, and complete vital signs on arrival. Laboratory variables included peripheral blood and pleural fluid analyses, while microbiological assessments comprised blood and pleural fluid cultures, along with molecular testing. Radiological variables included maximum effusion size, presence of loculations, mediastinal shift, and radiological complications (necrotizing pneumonia, bronchopleural fistula, pneumothorax, etc.).

Therapeutic data included type of PPE, initial antibiotic regimen, duration of intravenous and total antibiotic therapy, indication of pleural drainage, and admission to the pediatric intensive care unit (PICU), together with respiratory support (requirement, type, and duration). Outcome variables comprised length of hospital stay, duration of fever, complications, need for PPE-related readmission, time to radiological resolution, and long-term sequelae.

### 2.4. Outcomes of the Study

No single primary outcome was prioritized. Study outcomes comprised most of the therapeutic and outcome variables: admission to the PICU, requirement and duration of respiratory support, length of hospital stay, duration of fever, duration of intravenous and total antibiotic therapy, occurrence of complications, time to radiological resolution, need for PPE-related readmission, and presence of long-term sequelae.

### 2.5. Statistical Analysis

Descriptive statistics were reported as mean with standard deviation (SD) for continuous variables following a normal distribution, as median with interquartile range (IQR) for continuous variables not following a normal distribution, and as absolute numbers for categorical variables. Data normality was assessed using the Shapiro–Wilk test. Comparisons between groups were performed using Student’s *t* test or Mann–Whitney U test for continuous variables and χ^2^ test or Fisher’s exact test for categorical variables, as appropriate. Additional between-group comparisons were conducted to assess differences in management strategies and clinical outcomes between the interventional and conservative periods. Statistical significance was defined as a two-sided *p*-value < 0.05.

Clinical outcomes were compared between the two predefined temporal periods. Multivariate logistic regression analyses were performed to account for potential baseline differences in disease severity and reduce confounding between groups. Within the conservative period, additional analyses were conducted to identify factors associated with pleural drainage. Statistical analyses were performed using SPSS version 25.0 (IBM Corp., Armonk, NY, USA).

## 3. Results

### 3.1. Epidemiology and Clinical Features

**Figure 1:**
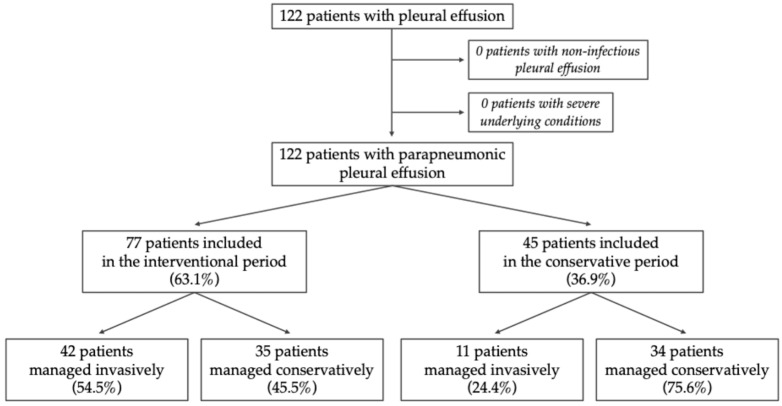
Flowchart illustrating patient inclusion and classification of parapneumonic pleural effusion according to management period and therapeutic approach.

A total of 122 children with radiologically confirmed PPE were included, and none fulfilled the defined exclusion criteria of non-infectious pleural effusion or severe uncontrolled underlying condition (Figure 1). From those, 62 patients (50.8%) were referred from non-tertiary centers. Cases were distributed across all study years (approximately 11 cases per year, ranging from 2 in 2020 to 20 in 2023) and throughout the year.

**Figure 2:**
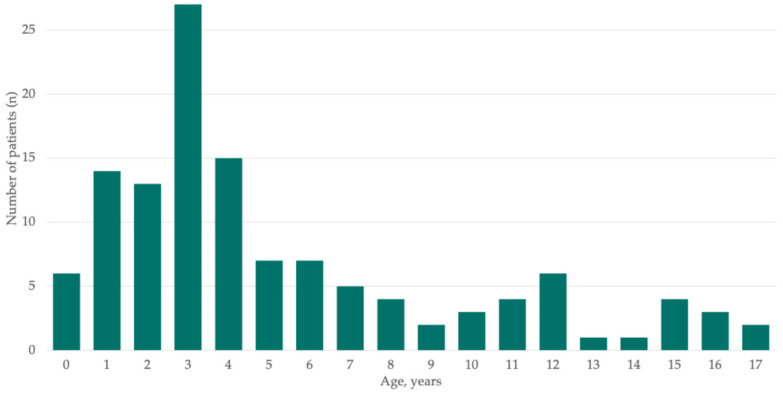
Age distribution of children with parapneumonic pleural effusion in the cohort (N = 122).

The median age at presentation was 4.1 years (IQR: 2.8–7.9), with cases spanning the entire pediatric age range (from 2 months to 17 years and 11 months; Figure 2). Sex distribution was equal, with 61 females (50%). Most patients were previously healthy, with only 2 cases (1.6%) classified as mildly immunocompromised (one case of sickle-cell disease and one case of immunoglobulin A deficiency). Complete immunization against *Streptococcus pneumoniae* had been administered to most patients (84.4% overall; 93.3% after the universal funding of the pneumococcal vaccine in Catalonia in 2016). Sixty-two patients (50.8%) were transferred from other hospitals, while the remaining 60 (49.2%) were admitted directly from the emergency department.

**Figure 3:**
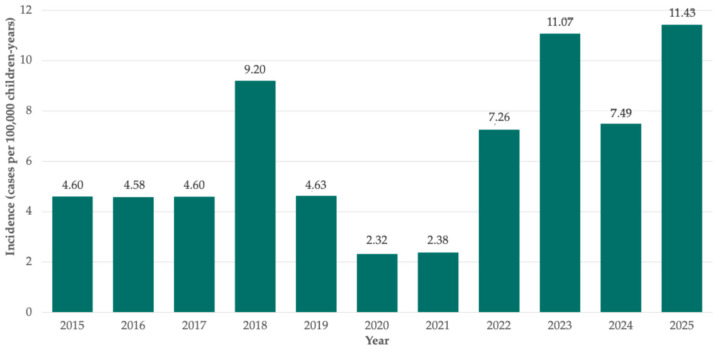
Incidence of parapneumonic pleural effusion during the study period in our setting.

The incidence of PPE showed marked temporal variation over the study period. Among patients not referred from other hospitals, the incidence rate of PPE remained relatively stable between 2015 and 2019, with around 4.6 cases per 100,000 children-years. A decline was then observed during the COVID-19 pandemic (2020–2021), followed by a pronounced post-pandemic rebound that has continued thereafter. Incidence peaked in 2025 at 11.43 cases per 100,000 children-years (Figure 3).

**Figure 4:**
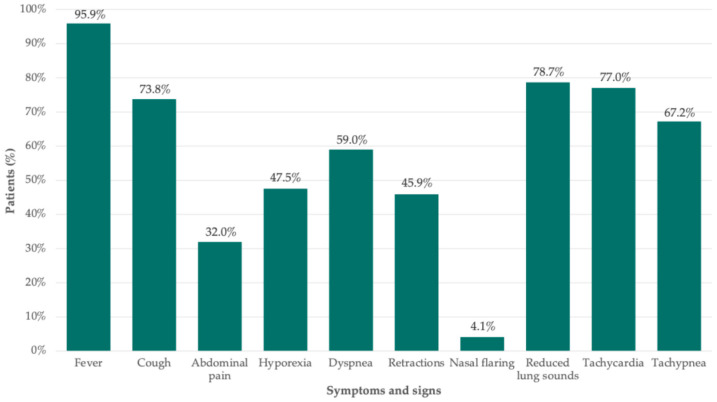
Clinical presentation of children with parapneumonic pleural effusion in the study cohort.

At presentation, fever was nearly universal (95.9%), whereas most patients also presented with respiratory symptoms such as cough (73.8%) and dyspnea (59.0%), together with clinical signs of respiratory distress, including reduced breath sounds (78.7%), retractions (45.9%), tachycardia (77.0%), and tachypnea (67.2%). One in three children (32.0%) also reported abdominal pain (Figure 4).

Regarding imaging modalities, both chest ultrasound and chest radiography were performed in all patients. Chest ultrasound was the most frequently used modality (median 4 examinations per patient, IQR: 2–5) and was particularly helpful for diagnosing and assessing PPE and cPPE, whereas chest radiography was performed at similar rates (median 4 examinations per patient, IQR: 2–5) and contributed to the overall diagnostic evaluation and assessment of potential complications.

Laboratory findings at admission revealed marked systemic inflammation, with elevated total leukocyte counts (median 18.15 × 10^9^/L, IQR: 12.8–23.4), neutrophil counts (median 13.25 × 10^9^/L, IQR: 8.8–18.3), and high C-reactive protein levels (median 245 mg/L, IQR: 127–338) in most patients. Other laboratory findings include a mean hemoglobin of 109 g/L (SD: 15), median platelet counts of 363 × 10^9^/L (IQR: 247.5–515) and a mean sodium level of 136 mEq/L (SD: 3). Pleural fluid analysis was performed in 87 patients (71.3%), with findings consistent with empyema in 43 cases (49.4% of those who underwent pleural fluid analysis).

A pathogen was identified in nearly half of the cases (49.2%), either through conventional cultures (blood or pleural fluid) or molecular techniques (blood, pleural fluid or nasopharyngeal swab). Blood cultures showed a low diagnostic yield (bacteremia rate: 18.0%), particularly among patients who had recently received antibiotics. Bacterial identification was significantly higher when pleural fluid cultures were obtained. Accordingly, despite the availability of new molecular techniques, overall bacterial identification rates have declined in recent years, as thoracocentesis has become less frequent in clinical practice.

**Figure 5:**
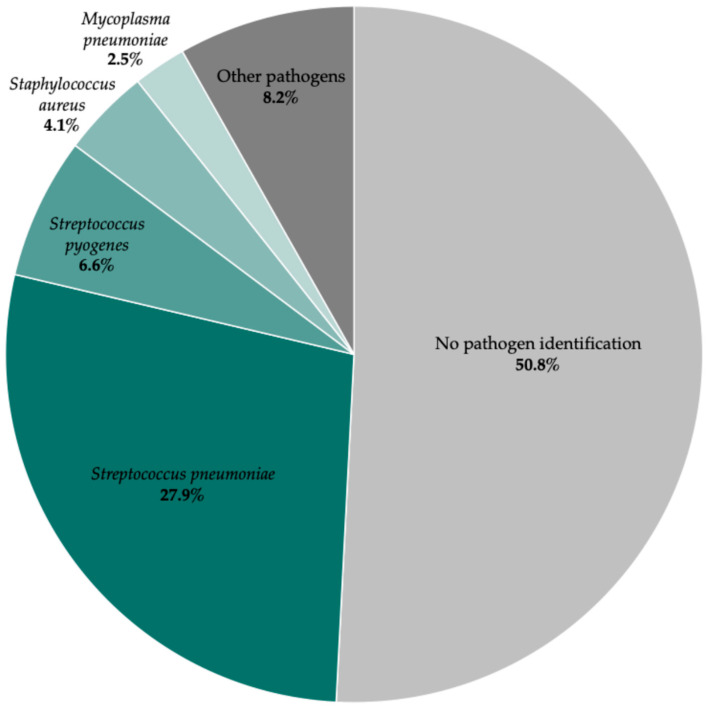
Microbiological etiology of parapneumonic pleural effusion cases in the study cohort.

Among culture-positive cases, the etiological profile was dominated by pyogenic bacteria, with *Streptococcus pneumoniae* as the leading pathogen (56.7%). Pneumococcal serotypes could not be specified as they were not determined in our setting. Other Gram-positive bacteria included *Streptococcus pyogenes* (13.3%), *Staphylococcus aureus* (8.3%), *Streptococcus intermedius* (3.3%), and *Staphylococcus epidermidis* (1.7%). Gram-negative and anaerobic bacteria were less common, with occasional isolation of *Fusobacterium necrophorum* (3.3%), *Haemophilus influenzae* (3.3%), *Enterobacter cloacae* (1.7%), and *Fusobacterium nucleatum* (1.7%). Increased detection of *Mycoplasma pneumoniae* (5.0%) was also observed in the last three years of the cohort, along with a single case of *Mycobacterium tuberculosis* (1.3%) (Figure 5).

During hospitalization, 78 patients (63.9%) required respiratory support, of whom 22 (28.2%) needed mechanical ventilation and 5 (6.4%) required intubation. Thirty-two patients (26.2%) were admitted to the PICU, most due to respiratory failure (68.8%), and the remainder due to sepsis or poor general condition (31.2%). The median duration of fever was 11 days (IQR: 7–17). PPE measured by ultrasound had a median maximal thickness of 2.5 cm (IQR: 1.5–3). A substantial proportion of patients (76, 62.3%) fulfilled biochemical and/or radiological criteria for cPPE, of whom 43 (56.6%) were diagnosed with empyema.

A total of 53 patients (43.4%) underwent pleural drainage, which was significantly more common in cPPE (59.2% vs. 17.4%, *p* < 0.01) and kept for a median of 4.5 days (IQR: 3–8.5). Fibrinolysis was used in 18 cases (34.0%) among those managed invasively. Video-assisted thoracoscopic surgery was only needed once (0.8%), in a teenage girl with a massive empyema not responding to other therapies.

The most common empiric antibiotic regimen consisted of a third-generation cephalosporin (ceftriaxone or cefotaxime) together with clindamycin (72.9%), while broader spectrum regimens such as piperacillin-tazobactam and vancomycin were reserved for critically ill or PICU-admitted patients (8.2%). Since 2023, empiric treatment with ampicillin has been increasingly used in stable, otherwise healthy children older than 24 months, accounting for 20.0% of cases in the last three years. The median duration of intravenous antibiotic therapy was 12 days (IQR: 8.5–18.5), and the median duration of total antibiotic therapy was 30 days (IQR: 23–38).

The most common complication was necrotizing pneumonia (26.4%), while pneumothorax was less frequent (7.3%) and occurred only as a procedure-related complication. There were 16 cases of pneumatocele (13.1%) and one case of an intrapulmonary abscess (0.8%), whereas no other pulmonary complications, such as bronchopleural fistula, were observed. As for non-pulmonary complications, 11 patients (9.0%) developed syndrome of inappropriate antidiuretic hormone secretion, 2 patients (1.6%) had concomitant acute appendicitis, and there was one case (0.8%) each of staphylococcal vertebral osteomyelitis, paroxysmal supraventricular tachycardia, and nephritic syndrome. The two patients (1.6%) with *Fusobacterium necrophorum* detection developed Lemierre’s syndrome. No cases of hemolytic-uremic syndrome cases were observed.

During outpatient follow-up, time to radiologic resolution was highly variable but generally prolonged (median 88 days, IQR: 44–184), and was significantly longer in patients with lung necrosis (median 110 days, *p* < 0.01). No major long-term sequelae were reported in any case. Only three patients (2.5%) required hospital readmission related to PPE, and no deaths occurred.

### 3.2. Outcome Comparison Between Management Periods

All 122 patients were included in this comparative analysis, with 77 managed during the interventional period and 45 during the conservative period (Table 1). Pleural drainage was performed more frequently during the interventional period (55% vs. 24%).

Baseline demographic characteristics were largely comparable between the two periods; however, patients admitted during the interventional period were significantly younger (3.8 vs. 5.0 years). Clinical presentation on arrival was also similar between periods, as rates of fever, abdominal pain, dyspnea, retractions, reduced breath sounds, hypoxemia, tachycardia, and need for oxygen therapy did not differ significantly, except for tachypnea, which was more common during the conservative period (60% vs. 80%).

Both initial and maximal pleural effusion thickness were comparable between groups, and no significant differences were observed in other imaging or laboratory variables. The number of chest ultrasounds performed was similar between periods, whereas significantly fewer chest radiographs were obtained in the conservative group (3 vs. 4). Rates of respiratory support and PICU admission were also comparable.

Patients managed during the conservative period experienced an overall more benign disease course, with shorter duration of intravenous antibiotic therapy (10 vs. 14.5 days, *p* < 0.01), duration of fever (9 vs. 12 days, *p* = 0.03), hospital stay (11 vs. 16 days, *p* < 0.01), and time to radiological resolution (62 vs. 102.5 days, *p* < 0.01). No significant differences were found in the incidence of lung necrosis or other complications.

To determine whether the observed differences in clinical outcomes were attributable to differences in baseline disease severity between periods, we performed a series of multivariate logistic regression analyses, adjusting for key clinical and radiological markers of severity at presentation. Separate models were constructed for three clinically relevant outcomes: PICU admission, need for respiratory support, and development of cPPE (Table 2). Across all models, management period was included as the main exposure variable, with four other additional relevant variables entered as covariates.

In the three multivariate models, overall performance was good. After adjustment for baseline severity, management period was not independently associated with PICU admission (adjusted OR: 1.57, 95% CI: 0.58–4.30, *p* = 0.38), need for respiratory support (adjusted OR: 1.24, 95% CI: 0.51–3.02, *p* = 0.63), or cPPE (adjusted OR: 0.95, 95% CI: 0.35–2.56, *p* = 0.92).

### 3.3. Factors Associated with Pleural Drainage Within the Conservative Period

A total of 45 patients with PPE were managed at our institution during the defined conservative management period (2023–2025). Among them, 11 patients (24.4%) underwent pleural drainage, while 34 (75.6%) were managed conservatively. Baseline demographic characteristics were comparable between groups, with no significant differences found in age and sex distribution, immunization status, symptoms at presentation, vital signs on arrival, or initial oxygen requirements (Table 3).

Radiological findings differed significantly between groups. Patients who underwent drainage had a greater initial and maximum pleural effusion thickness compared with those managed without drainage (both *p* < 0.01) and more frequently exhibited mediastinal shift (64% vs. 6%, *p* < 0.01). In contrast, no statistically significant differences were observed in laboratory variables.

Markers of clinical severity at presentation were more frequent in the drainage group, including higher rates of respiratory support (91% vs. 47%, *p* = 0.01) and PICU admission (73% vs. 6%, *p* < 0.01). No significant differences could be demonstrated in the proportion of cPPE.

Although microbiological identification was more frequent in the pleural drainage group (64% vs. 26%, *p* = 0.03), no differences were observed in the rate of associated bacteriemia or the proportion of pneumococcal etiology.

Patients undergoing drainage required more imaging studies, including a higher number of chest ultrasounds (4 vs. 3, *p* = 0.02) and chest radiographs (5 vs. 2, *p* < 0.01). They also showed a more prolonged clinical course, with longer antibiotic therapy (39 vs. 23.5 days, *p* < 0.01), longer duration of respiratory support (2 vs. 0 days, *p* < 0.01), and longer hospital stay (18 vs. 9.5 days, *p* < 0.01). Complications such as necrotizing pneumonia were also more frequent (64% vs. 15%, *p* < 0.01), although no deaths or long-term sequelae were observed in either group.

## 4. Discussion

### 4.1. Characterization and Temporal Changes in Pediatric Parapneumonic Pleural Effusion

The clinical characterization of pediatric PPE cases in our cohort is largely consistent with previously reported series. Most cases occurred in children younger than six years of age, reinforcing the observation that PPE predominantly affects younger children. The median age in our cohort (4.1 years) was comparable to previous series (4.0–5.0 years) [3,12,14,25,26]. Clinical presentation was dominated by fever and typical respiratory features, such as cough, dyspnea, retractions, and decreased breath sounds. Most patients exhibited marked laboratory abnormalities, including substantial leukocytosis and elevated C-reactive protein levels, confirming the hyperinflammatory profile of PPE, as well as variable radiological findings, consistent with previous series [3,25,26].

The proportion of cPPE in our cohort (62.3%) was higher than that reported in many previous pediatric series [1,16,25,27]. This likely reflected our role as a tertiary referral center, which inherently introduces selection and referral bias. In our setting, more severe or clinically complex cases are preferentially transferred from other hospitals, which resulted in a significantly higher proportion of cPPE cases among transferred patients compared with those primarily managed at our institution (77% vs. 49%, *p* < 0.01). As a result, the study population may not be fully representative of the overall spectrum of pediatric PPE, with underrepresentation of milder, uncomplicated cases and an overestimation of disease severity, proportion of cPPE, indication for invasive interventions, and resource utilization in our cohort.

To minimize referral bias, the yearly incidence of PPE was calculated using only primary patients. In this context, our data are consistent with other series, showing an increase in PPE cases in recent years [3,12,14,25,26,27], which in our setting presented as a sustained rise in cases in the post-pandemic period, reaching approximately 7–11 cases per 100,000 children-years. These findings, coupled with the reported growing burden of cPPE cases [9,10,11,12], indicate that PPE continues to represent a significant complication of pneumonia with a substantial healthcare impact.

The presence of bacteriemia in our cohort (18.0%) was comparable to that reported in the current literature (9.3–19.7%) [12,25,26,27]. In our cohort, bacteremia was not associated with increased clinical severity or complications, limiting its prognostic utility.

The etiological distribution observed in our cohort did not differ substantially from that described in other European studies [14,25,26]. *Streptococcus pneumoniae* was the most common pathogen, followed by *Streptococcus pyogenes* and *Staphylococcus aureus*. Notably, *Staphylococcus aureus* and *Haemophilus influenzae* were only identified in children younger than 24 months, whereas *Mycoplasma pneumoniae*, coagulase-negative streptococci, and *Fusobacterium* tended to occur in children older than 10 years, a finding that also corresponds to present literature [24,28,29]. Although reported in some settings [12,25], no Gram-negative enterobacteria such as *Pseudomonas aeruginosa* or *Klebsiella pneumoniae* were isolated in our population.

Despite this relatively homogeneous demographic and etiological profile, clinical evolution within the cohort was highly heterogeneous. Approximately two-thirds of patients met criteria for cPPE, a similar proportion required respiratory support, and more than one quarter were admitted to the PICU. Pleural drainage was required in 43.4% of patients, while the use of fibrinolytic agents and surgical techniques was limited. Overall, uncomplicated PPE cases with rapid resolution under medical therapy alone coexisted with severe presentations requiring invasive procedures and a prolonged hospital stay.

As for empiric antimicrobial therapy, the most commonly used regimen was a combination of clindamycin and ceftriaxone or cefotaxime, whereas the combination of vancomycin and piperacillin-tazobactam was reserved for septic or PICU-admitted patients. As previously suggested by other authors [30,31], a progressive shift toward ampicillin was observed in the last three years of the study.

Finally, the overall burden of complications in our cohort was relatively limited. Necrotizing pneumonia was the most frequent pulmonary complication, whereas pneumothorax occurred exclusively as an iatrogenic event, and other pulmonary complications were rare. Extrapulmonary complications were also infrequent. Taken together, and in comparison with other published cohorts [3,12,23,25], our population appears to be characterized by a low prevalence of underlying comorbidities, few severely ill patients, and generally favorable outcomes. Our patients required few intubations, had limited need for surgery, and no mortality was observed. These findings are consistent with the fact that, despite the potential severity of PPE, most previously healthy children can experience good clinical outcomes when managed with an appropriate and individualized approach.

### 4.2. Comparison of Management Strategies and Factors Associated with Pleural Drainage

A key finding of the present study is that, when comparing PPE cases across the two pre-established periods, the shift from a predominantly invasive to a predominantly conservative management strategy was not associated with worse clinical outcomes. Furthermore, some important outcomes, such as the duration of intravenous antibiotic therapy and the length of hospital stay, were significantly reduced during the conservative period.

Unadjusted comparisons between management periods revealed that patients managed during the conservative period (2023–2025) were significantly older. These differences raise the possibility of selection bias, and crude outcome comparisons between periods should therefore be interpreted with caution. To address this limitation, multivariate analyses were performed to adjust for baseline disease severity at presentation. These analyses showed that the management period was not independently associated with PICU admission, need for respiratory support, or development of cPPE. These findings suggest that our temporal grouping did not independently predict disease severity or outcomes and support the validity of the present study.

However, the temporal definition of the study periods may be a limitation. Although the division into an interventional and a conservative period reflects a shift in clinical practice at our institution, the relatively short duration of the latter period (3 years) could have introduced imbalances in patient characteristics and limited the stability of comparisons. Moreover, it coincided with the post-COVID-19 era, during which substantial epidemiological changes have been reported. These external factors may have influenced both the case mix and clinical outcomes independently of management strategy, potentially confounding the observed differences between periods.

The shift toward a conservative strategy was also accompanied by a more selective use of invasive procedures, including thoracocentesis, pleural drainage, and fibrinolysis, without evidence of worse outcomes among appropriately selected patients. This selective approach may reduce procedure-related complications such as iatrogenic pneumothorax, patient and family discomfort, and resource utilization, while preserving clinical safety. Importantly, no increase in severe complications or duration of hospitalization was observed during the conservative period after accounting for baseline differences.

To further explore how pleural drainage was indicated across the severity spectrum of our population, outcomes were analyzed among patients managed during the conservative period according to whether drainage was performed. This analysis showed that pleural drainage was preferentially used in patients presenting with more severe radiological involvement, alongside with rates of higher respiratory support and PICU admission. These findings support the notion that pleural drainage was mainly reserved for children with significantly more severe disease and greater clinical compromise.

Patients who underwent pleural drainage also experienced a more prolonged clinical course, with longer antibiotic therapy, longer duration of respiratory support, and longer hospital stay, as well as a higher frequency of lung necrosis. These differences should be interpreted in the context of confounding by indication. In routine clinical practice, pleural drainage is more likely to be performed in patients with more severe disease at presentation, and this same baseline severity may independently drive a more complicated clinical course. This well-recognized limitation is inherent to retrospective observational studies evaluating therapeutic interventions.

Another notable finding is that the presence of cPPE was not independently associated with the decision to perform pleural drainage during the conservative period. Instead, drainage appeared to be guided primarily by clinical compromise. This contrasts with many current clinical guidelines, which consider cPPE an absolute indication of pleural drainage [4]. Despite this more selective approach, no evidence of worse overall outcomes was observed in the cohort managed during the conservative period.

Nevertheless, exclusive medical therapy should not be interpreted as a universal strategy. Pleural drainage remains essential in children with severe pleural disease, such as large effusions causing mediastinal shift, significant respiratory compromise, or poor response to medical therapy alone. The absence of worse outcomes in the conservative period likely reflects improved patient selection rather than a generalized reduced role for invasive interventions.

In conclusion, these findings seem to support a tailored, severity-driven approach to pediatric PPE management. While pleural drainage remains justified in children with significant clinical and/or radiological impact, medical therapy alone appears to be safe in selected patients, provided that close monitoring is ensured.

### 4.3. Clinical Implications, Strengths, Limitations, and Future Directions

The findings of this study have several relevant implications for the clinical management of pediatric PPE. A more selective use of pleural drainage may contribute to improved patient safety by reducing exposure to invasive procedures, including pleural drainage, thoracic surgery, and the need for sedation of general anesthesia.

From a healthcare system perspective, a more conservative and selective approach may lead to significant cost savings by decreasing the use of procedural resources, limiting referrals to tertiary-care centers, and reducing the need for surgical or interventional radiology teams. Consequently, these findings may contribute to a more standardized and equitable approach to care, helping to reduce variability in clinical practice and promote more consistent decision-making across centers.

This study has important strengths. It includes a well-characterized cohort of pediatric patients with PPE managed in a real-world clinical setting, with comprehensive clinical, laboratory, and radiological data. The temporal comparison between management strategies provides insight into changes in clinical practice over time, while the use of multivariate analyses to adjust for baseline severity strengthens the robustness of the study findings.

Nevertheless, several limitations should be acknowledged. The retrospective, single-center design limits causal inference and may reduce generalizability to other settings with different patient populations or management protocols. Temporal comparisons between management periods are inherently subject to confounding, as changes in clinical practice may coincide with shifts in referral patterns, disease epidemiology, or diagnostic thresholds. Although multivariate adjustment was used to account for baseline severity differences, residual confounding cannot be fully excluded. In addition, the relatively modest sample size may have limited the statistical power to detect smaller differences in less frequent outcomes.

Future research should focus on prospective validation of these findings in multicenter cohorts. Randomized studies comparing invasive and conservative strategies in well-defined risk groups would be valuable to confirm the safety and effectiveness of severity-guided management approaches.

## 5. Conclusions

PPE remains a relevant complication of pediatric pneumonia, representing a substantial healthcare burden despite generally favorable outcomes. In our cohort, most cases occurred in young children and were predominantly caused by *Streptococcus pneumoniae*, with clinical and microbiological features comparable to previously reported series. Although a considerable proportion of patients developed cPPE or required respiratory support, severe complications and mortality were uncommon.

A major finding of this study is that the transition from a predominantly interventional to a more conservative management strategy was not associated with worse clinical outcomes after adjustment for baseline severity. During the conservative period, invasive procedures were used more selectively without evidence of increased complications, delayed recovery, or adverse outcomes.

These results support a severity-guided approach to pediatric PPE management. Conservative treatment with close monitoring appears to be safe in carefully selected patients without markers of severe pleural disease, while invasive interventions remain appropriate for patients with significant radiological involvement or clinical compromise. Prospective and multicenter studies are warranted to further define the optimal indications for pleural drainage in pediatric PPE.

## Figures and Tables

**Table 1:** Comparison of variables between study periods. μ: mean. SD: standard deviation. M: median. IQR: interquartile range.

Variables	Patients in the Interventional Period (2015–2022; N = 77)	Patients in the Conservative Period (2023–2025; N = 45)	*p*-Value
**Epidemiological variables**			
Female sex, n (%)	38 (49)	23 (51)	0.85
Age (years), M (IQR)	3.8 (2.3–6.9)	5.0 (3.2–11.4)	<0.01
Anti-pneumococcal vaccination, n (%)	60 (78)	43 (96)	<0.01
Viral coinfection, n (%)	10 (13)	11 (24)	0.11
**Clinical variables on arrival**			
Fever, n (%)	74 (96)	43 (96)	1.00
Abdominal pain, n (%)	28 (36)	11 (24)	0.17
Dyspnea, n (%)	44 (57)	28 (62)	0.58
Chest wall retractions, n (%)	38 (49)	18 (40)	0.32
Reduced breath sounds, n (%)	61 (79)	35 (78)	0.85
Tachycardia, n (%)	61 (79)	33 (73)	0.46
Tachypnea, n (%)	46 (60)	36 (80)	0.02
Need for oxygen therapy, n (%)	23 (30)	11 (24)	0.52
**Radiological variables**			
Initial effusion thickness (cm), M (IQR)	1.6 (1.1–2.5)	1.6 (1.0–2.6)	0.44
Maximum effusion thickness (cm), M (IQR)	2.5 (1.8–3.4)	2.3 (1.5–3.0)	0.08
Mediastinal shift, n (%)	24 (31)	9 (20)	0.18
**Initial laboratory variables**			
Leukocyte count (10^9^/L), M (IQR)	18.00 (13.45–23.70)	18.30 (12.45–23.45)	0.21
Neutrophil count (10^9^/L), M (IQR)	13.30 (9.35–18.55)	13.10 (7.70–18.25)	0.16
C-reactive protein (mg/L), M (IQR)	279 (140–340)	220 (95–335)	0.22
Hemoglobin level (g/L), μ (SD)	107 (14)	111 (17)	0.05
Platelet count (10^9^/L), M (IQR)	354.0 (235.5–470.0)	410.0 (276.0–576.5)	0.06
Sodium level (mEq/L), μ (SD)	136.3 (3.1)	136.5 (3.5)	0.27
**Management variables**			
Complicated pleural effusion, n (%)	52 (68)	25 (56)	0.19
Pleural fluid analysis, n (%)	66 (86)	22 (49)	<0.01
Pleural drainage, n (%)	42 (55)	11 (24)	<0.01
Fibrinolysis, n (%)	17 (22)	1 (2)	<0.01
Respiratory support, n (%)	52 (68)	26 (58)	0.28
Intensive care unit admission, n (%)	22 (29)	10 (22)	0.44
**Microbiological variables**			
Bacteriemia, n (%)	15 (19)	7 (16)	0.59
Microbiological identification, n (%)	44 (57)	16 (36)	0.02
Pneumococcal etiology, n (%)	26 (59)	8 (50)	0.53
**Resource use and evolution variables**			
Ultrasounds performed (n), M (IQR)	4 (2–5)	3 (2–5)	0.93
Chest X-rays performed (n), M (IQR)	4 (3–5)	3 (1.5–4)	0.02
Intravenous antibiotics (days), M (IQR)	14.5 (10–20)	10 (6–15)	<0.01
Oral antibiotics (days), M (IQR)	15 (11–20)	14 (7–18)	0.64
Total antibiotics (days), M (IQR)	31 (25–38)	27 (19.5–37.5)	0.10
Fever length (days), M (IQR)	12 (8–18)	9 (5.5–14)	0.03
Respiratory support length (days), M (IQR)	2 (0–5)	1 (0–2)	0.08
Hospital stay (days), M (IQR)	16 (12–22)	11 (7–17.5)	<0.01
Lung necrosis, n (%)	20 (26)	12 (27)	0.93
Radiologic resolution (days), M (IQR)	102.5 (62–194)	62 (23.5–145)	<0.01

**Table 2:** Multivariate logistic regression models for three clinically relevant outcomes, with management period, age, chest wall retractions, maximal pleural effusion thickness, and mediastinal shift as exposure variables. OR: odds ratio. CI: confidence interval.

**(A)**	**First Model: Pediatric Intensive Care Unit Admission**			
**Variable**	**Coefficient**	**Standard Error**	***p*-Value**	**OR (95% CI)**
Interventional period (%)	0.454	0.512	0.376	1.574 (0.577–4.296)
Age (years)	0.145	0.052	**0.006**	1.156 (1.043–1.282)
Chest wall retractions (%)	0.974	0.477	**0.041**	2.648 (1.040–6.742)
Maximal pleural effusion thickness (cm)	−0.047	0.137	0.734	0.955 (0.730–1.248)
Mediastinal shift (%)	1.433	0.508	**0.005**	4.192 (1.550–11.338)
Constant	−3.101	0.730	**0.000**	
**Model fit:**	**Chi-square** = 21.718	**df** = 5	***p*-Value** = **0.001**	
**(B)**	**Second model: Need for respiratory support**			
**Variable**	**Coefficient**	**Standard error**	***p*** **-value**	**OR (95% CI)**
Interventional period (%)	0.216	0.454	0.634	1.241 (0.510–3.020)
Age (years)	0.022	0.051	0.676	1.022 (0.924–1.130)
Chest wall retractions (%)	1.862	0.466	**0.000**	6.438 (2.584–16.042)
Maximal pleural effusion thickness (cm)	0.038	0.162	0.816	1.039 (0.756–1.427)
Mediastinal shift (%)	1.769	0.655	**0.007**	5.865 (1.626–21.152)
Constant	−0.884	0.602	0.142	
**Model fit:**	**Chi-square** = 31.782	**df** = 5	***p*-Value** = **0.000**	
**(C)**	**Third model: Complicated parapneumonic pleural effusion**			
**Variable**	**Coefficient**	**Standard error**	***p*** **-value**	**OR (95% CI)**
Interventional period (%)	−0.052	0.506	0.918	0.950 (0.352–2.561)
Age (years)	−0.303	0.068	**0.000**	0.739 (0.646–0.844)
Chest wall retractions (%)	1.020	0.493	**0.039**	2.772 (1.055–7.282)
Maximal pleural effusion thickness (cm)	0.824	0.262	**0.002**	2.280 (1.365–3.806)
Mediastinal shift (%)	1.535	0.723	**0.034**	4.642 (1.125–19.153)
Constant	−0.381	0.732	0.603	
**Model fit:**	**Chi-square** = 55.129	**df** = 5	***p*-Value** = **0.000**	

**Table 3:** Comparison of variables between patients managed with and without pleural drainage during the conservative period (2023–2025). μ: mean. SD: standard deviation. M: median. IQR: interquartile range.

Variables	Patients Without Pleural Drainage (2023–2025; N = 34)	Patients with Pleural Drainage (2023–2025; N = 11)	*p*-Value
**Epidemiological variables**			
Female sex, n (%)	17 (50)	6 (55)	0.79
Age (years), M (IQR)	4.7 (2.9–10.1)	8.8 (3.6–15.8)	0.08
Anti-pneumococcal vaccination, n (%)	34 (100)	9 (82)	0.06
Viral coinfection, n (%)	8 (24)	3 (27)	1.00
**Clinical variables on arrival**			
Fever, n (%)	33 (97)	10 (91)	0.43
Abdominal pain, n (%)	8 (24)	3 (27)	1.00
Dyspnea, n (%)	21 (62)	7 (64)	1.00
Chest wall retractions, n (%)	13 (38)	5 (45)	0.67
Reduced breath sounds, n (%)	25 (74)	10 (91)	0.41
Tachycardia, n (%)	24 (71)	9 (82)	0.70
Tachypnea, n (%)	26 (76)	10 (91)	0.42
Need for oxygen therapy, n (%)	7 (21)	4 (36)	0.42
**Radiological variables**			
Initial effusion thickness (cm), M (IQR)	1.2 (0.8–2.3)	3.0 (1.8–4.0)	<0.01
Maximum effusion thickness (cm), M (IQR)	1.9 (1.4–2.5)	3.5 (3.0–4.0)	<0.01
Mediastinal shift, n (%)	2 (6)	7 (64)	<0.01
**Initial laboratory variables**			
Leukocyte count (10^9^/L), M (IQR)	18.50 (12.50–23.50)	16.90 (8.20–21.30)	0.33
Neutrophil count (10^9^/L), M (IQR)	11.55 (8.00–18.30)	13.50 (6.50–18.20)	0.39
C-reactive protein (mg/L), M (IQR)	180 (89–340)	250 (186–295)	0.37
Hemoglobin level (g/L), μ (SD)	111 (16)	113 (22)	0.40
Platelet count (10^9^/L), M (IQR)	422.0 (286.0–595.0)	356.0 (116.0–536.0)	0.10
Sodium level (mEq/L), μ (SD)	136.5 (2.7)	136.6 (5.5)	0.24
**Management variables**			
Complicated pleural effusion, n (%)	16 (47)	9 (82)	0.08
Respiratory support, n (%)	16 (47)	10 (91)	0.01
Intensive care unit admission, n (%)	2 (6)	8 (73)	<0.01
**Microbiological variables**			
Bacteriemia, n (%)	4 (12)	3 (27)	0.34
Microbiological identification, n (%)	9 (26)	7 (64)	0.04
Pneumococcal etiology, n (%)	4 (44)	4 (57)	1.00
**Resource use and evolution variables**			
Ultrasounds performed (n), M (IQR)	3 (2–4)	4 (3–7)	0.02
Chest X-rays performed (n), M (IQR)	2 (1–3)	5 (4–6)	<0.01
Intravenous antibiotics (days), M (IQR)	8 (5–14)	26 (18–30)	<0.01
Oral antibiotics (days), M (IQR)	14 (14–19)	24.2 (8.4)	<0.01
Total antibiotics (days), M (IQR)	23.5 (19–31)	39 (30–56)	<0.01
Fever length (days), M (IQR)	9.5 (6–13)	7 (5–27)	0.32
Respiratory support length (days), M (IQR)	0 (0–2)	2 (1–6)	<0.01
Hospital stay (days), M (IQR)	9.5 (6–16)	18 (16–31)	<0.01
Lung necrosis, n (%)	5 (15)	7 (64)	<0.01
Radiologic resolution (days), M (IQR)	50 (18–119)	153 (33–247)	0.06

## Data Availability

The data could be shared by the corresponding author upon reason- able request.

## Abbreviations

The following abbreviations are used in this manuscript:

CI	Confidence interval
cPPE	Complicated parapneumonic pleural effusion
IQR	Interquartile range
OR	Odds ratio
PPE	Parapneumonic pleural effusion
SD	Standard deviation
